# DAMPs and PAMPs in the Perioperative Period: Danger Signaling, Immune Dysfunction, and Oncologic Implications

**DOI:** 10.3390/medicina62091807

**Published:** 2026-09-19

**Authors:** Hector Katifelis, Theofania Lappa, Sofia Poulopoulou, Maria Gazouli

**Affiliations:** 1 Department of Anaesthesiology, General Oncology Hospital of Athens “Saint Savvas” 171 Alexandras Ave., 11522 Athens, Greece; spoulopoulou@yahoo.com; 2 Laboratory of Biology, Department of Basic Medical Sciences, Medical School, National and Kapodistrian University of Athens, Michalakopoulou 176, 11527 Athens, Greece; t.lappa@med.uoa.gr (T.L.); mgazouli@med.uoa.gr (M.G.)

**Keywords:** DAMPs, PAMPs, cancer surgery, transfusion, perioperative medicine, danger signals, onco-anesthesia

## Abstract

*Background and Objectives*: The perioperative period is characterized by marked biological stress responses that extend beyond direct tissue injury. Innate immune activation during surgery is largely driven by molecular danger signals. Surgical trauma, ischemia–reperfusion injury, blood transfusions, mechanical ventilation, and perioperative infections are critical events that result in the release of danger signals: damage-associated molecular patterns (DAMPs) and pathogen-associated molecular patterns (PAMPs). This narrative review examines perioperative DAMP and PAMP sources, their molecular recognition pathways, and their clinical and oncological significance. *Materials and Methods*: A literature search was conducted across PubMed/MEDLINE, Scopus, and Web of Science databases for English-language articles published up to July 2026. Search terms included DAMPs, PAMPs, perioperative, surgical stress, cancer surgery, innate immunity, PRRs, TLRs, inflammasome, HMGB1, mtDNA, perioperative immunosuppression, and anesthesia. *Results:* Danger signals interact with pattern-recognition receptors, including Toll-like receptors and inflammasome pathways, driving sterile inflammation, immune dysregulation, and postoperative organ injury. Rather than initiating these cascades, anesthetics and opioids act as modulators. In cancer surgery, heightened danger signaling and temporary immunosuppression may compromise host defenses during a vulnerable window. *Conclusions*: Circulating DAMPs and potentially selected PAMP-related markers warrant further investigation as biomarkers for perioperative risk stratification. Overcoming translational barriers will require standardized assays, validation of biomarker signatures, and integration of artificial intelligence-driven molecular profiling to advance personalized onco-anesthesia strategies.

## 1. Introduction

Surgical intervention induces a complex biological stress response that drives considerable immune, metabolic, and neuroendocrine alterations. In the perioperative setting, such systemic perturbations shape short-term recovery, systemic inflammatory responses, and ultimately clinical outcomes [1]. Particularly in cancer surgery, where surgical resection continues to be the foundation of curative treatment for many solid malignancies [2], these perioperative responses have attracted attention due to their influence on immediate postoperative recovery and long-term oncologic trajectory. Due to ever-growing global surgical demand, with cancer surgery alone projected to approach 14 million patients annually by 2040 [3], understanding the precise pathways that drive perioperative immune activation becomes crucial.

A key component of these responses lies in the innate immune system, which is equipped with pattern recognition receptors (PRRs), an evolutionarily conserved system of sensors that detect molecular signals of danger. Such signals arise from invading pathogens, referred to as pathogen-associated molecular patterns (PAMPs), and from endogenous molecules released by stressed or injured cells, known as damage-associated molecular patterns (DAMPs) [4]. Typically, the perioperative setting includes injury in the absence of infection. In these cases, DAMPs represent the predominant drivers of sterile inflammation. However, PAMPs can also be present when infection, microbial translocation, or barrier disruption occur.

Despite growing recognition of the importance of these pathways, DAMP and PAMP signaling has not yet been incorporated into perioperative and anesthetic practice. An in-depth understanding of how these danger signals are generated, sensed, and translated into clinical effects could provide a mechanistic framework that links surgical stress to immune dysregulation and cancer progression. Recent advances in the emerging field of onco-anesthesia underscored the importance of understanding how perioperative management could influence immune function and long-term oncologic outcomes [5].

These observations suggest that perioperative danger signaling may form a central biological axis through which surgical injury reshapes host immunity and even affects tumor behavior.

While previous reviews on the perioperative period have focused primarily on sterile inflammation caused by DAMPs and on perioperative anesthesiologic care [6], this review expands this framework to several clinically relevant areas. First, it integrates DAMPs and PAMPs into a unified risk-signaling model during the perioperative period, rather than treating aseptic and microbial signals separately. Second, it places particular emphasis on cancer surgery and the transient state of immune vulnerability during the perioperative period. Third, it examines blood transfusion as a possible “second hit” and identifies anesthetics and opioids as modulators, rather than primary drivers, of risk signaling. Fourth, it provides a critical evaluation of the available human biomarker data, the limitations of assays and sampling, and the translational potential of complex molecular signatures, including artificial intelligence (AI)-assisted molecular profiling. Finally, it examines how perioperative strategies may influence these processes, highlighting opportunities for translational research and clinical optimization.

## 2. Materials and Methods

A literature search was conducted from December 2025 through July 2026 in PubMed/MEDLINE, Scopus and Web of Science to identify relevant studies examining DAMPs and PAMPs in the perioperative setting. Approximately 250 records were initially identified across the three databases. Following assessment of relevance to the objectives and scope of the review, the literature was selectively synthesized, with 71 publications cited in the final narrative review. The search strategy included combinations of the following terms: DAMPs, PAMPs, alarmins, perioperative, surgical stress, cancer surgery, innate immunity, PRRs, TLRs, inflammasome, HMGB1, mtDNA, perioperative immunosuppression, and anesthesia. Representative search combinations included (“DAMPS” OR “damage-associated molecular patterns” OR alarmins) AND (perioperative OR surgery OR surgical stress OR anesthesia) and (“PAMPS” OR “pathogen-associated molecular patterns”) AND (perioperative OR surgery OR infection OR microbial translocation). The final search update was performed in July 2026.

Articles were considered eligible when they addressed the biological mechanisms, perioperative sources, immune signaling, clinical consequences or oncological implications of DAMPs/PAMPs and were relevant to the scope of this narrative review. Primary experimental, translational, observational and clinical studies, as well as relevant review articles, were considered. Only English-language publications were included. Studies were selected based on their relevance to the objectives and scope of the review. Reference lists of key articles were manually screened to identify additional relevant studies. Because this was a narrative review, formal systematic-review methodology, formal risk-of-bias assessment, and evidence grading were not applied.

## 3. Origin of DAMPs/PAMPs in the Perioperative Period

The perioperative period is a critical phase characterized by the release of endogenous and, to a lesser extent, exogenous danger signals shaping the immune response to surgical stress. Surgical trauma is a primary source of DAMP release. These molecules can be either actively secreted by immune cells and stressed host cells or passively released directly from damaged cells into the extracellular environment [7,8].

### 3.1. Tissue Trauma and DAMP Release

Surgical incision and tissue manipulation cause cellular disruption, resulting in the release of high mobility group box 1 (HMGB1), histones, extracellular adenosine triphosphate (ATP), S100A8/A9 proteins, heat shock proteins (HSPs), and mitochondrial DNA (mtDNA). At the same time, extracellular matrix degradation releases molecules including biglycan and low-molecular-weight hyaluronan, which further boost inflammatory signaling [9]. Ischemia–reperfusion injury, observed in vascular, transplant, and cardiac surgery, enhances DAMP generation via ATP depletion, production of reactive oxygen species (ROS), and activation of regulated cell death including pyroptosis, necroptosis, and ferroptosis. These perioperative DAMP responses have also been demonstrated in human surgical cohorts [10,11].

Cardiopulmonary bypass also triggers systemic inflammation. This occurs via blood interaction with artificial surfaces and ischemia–reperfusion mechanisms. Studies on human cardiac surgery have demonstrated perioperative increases in circulating DAMPs, including HMGB1, histones, and free mitochondrial DNA [12,13]. Blood transfusion represents another important source, as storage-related cellular damage leads to the release of free heme, cell-free DNA (cfDNA), ATP, HMGB1, and microparticles, which have been implicated in transfusion-related acute lung injury and transfusion-related immunomodulation.

Mechanical ventilation may also play a role, particularly when high tidal volumes are used. Experimental evidence indicates that injurious mechanical ventilation can increase extracellular ATP in the alveolar compartment and contribute to lung inflammation [14]. 

### 3.2. Exogenous Danger Signals: PAMPs

In contrast to DAMPs, which arise from host tissue injury, PAMPs are exogenous microbial motifs that are normally absent from sterile tissues and the bloodstream. Patients face PAMP exposure through multiple perioperative routes. Common sources include contaminated surgical equipment or wounds, pre-existing sepsis, bacterial translocation across breached epithelial barriers, and systemic spread from distant infections. Representative perioperative PAMPs include lipopolysaccharide (LPS), peptidoglycan, lipoteichoic acid, flagellin, unmethylated CpG bacterial DNA, and fungal β-glucans, which are detected by distinct families of pattern-recognition receptors [15,16]. However, direct perioperative evidence for PAMPs is substantially weaker than the evidence for DAMPs; colonization or barrier disruption should not be equated with microbial translocation, circulating microbial products, infection, or downstream PRR activation.

Perioperative PAMP signaling does not require overt bacteremia or culture-positive infection. In patients undergoing major abdominal or oncologic surgery, bowel preparation failure, mucosal barrier disruption, ischemia, tissue manipulation, and tumor-associated dysbiosis may facilitate translocation of bacteria or bacterial products into the circulation. Thus, perioperative PAMP exposure may reflect not only frank infection, but also the systemic dissemination of endotoxin or microbial DNA in the setting of barrier dysfunction. This concept is particularly relevant in gastrointestinal malignancies, where bacterial translocation has been increasingly linked to inflammation, treatment response, and tumor biology [15].

The oral cavity can act as another source of perioperative PAMPs. Periodontitis and poor oral hygiene are associated with chronic exposure to microbial ligands, intermittent bacteremia, and postoperative infectious complications. Meta-analytic and clinical data suggest that periodontitis is associated with a higher risk of postoperative complications, while perioperative oral management may reduce postoperative pneumonia and surgical-site infection. This is especially important in patients undergoing major gastrointestinal and thoracic cancer surgery. Such observations support the idea that chronic oral infection may act as a remote perioperative source of PAMPs even when the operative field is anatomically distant [17,18].

From an oncologic perspective, the relevance of PAMPs may extend beyond their established role in infection. Microbial products can activate TLR and NF-κB-dependent pathways, modulate the tumor microenvironment, and potentially promote cancer progression. This concept has been explored in gastrointestinal cancers, where bacterial translocation and tumor-associated microbes such as *Fusobacterium nucleatum* have been linked to inflammation, immune evasion, chemoresistance, and poor prognosis. Although direct perioperative evidence is limited, these observations offer biological plausibility for a role of perioperative PAMP exposure in shaping postoperative immune responses and possibly oncologic outcomes [15,19].

Finally, PAMPs should not be viewed in isolation, because they may act synergistically with DAMPs released during surgery. Tissue injury provides endogenous ligands, whereas infection, colonization, or microbial translocation provide exogenous ligands that converge on overlapping PRR pathways. The net effect may be amplification rather than simple addition of inflammatory signaling, making combined DAMP/PAMP exposure particularly important in complex oncologic surgery [6,16].

### 3.3. Transfusion as a Second Hit: DAMP-Mediated Immunomodulation in the Perioperative Setting

The role of transfusion in cancer surgery is complicated. Observational studies have associated perioperative allogeneic blood transfusion with immune modulation and adverse oncologic outcomes, including recurrence and reduced survival; however, residual confounding prevents firm causal conclusions [20,21,22]. In parallel, transfusions are a known source of DAMPs, especially mtDNA, which has been found in multiple blood products (fresh frozen plasma, FFP, red blood cells, RBCs, and platelet concentrates, PCs). Thus, in addition to surgery itself being a source of DAMPs, transfusion may represent an independent source of DAMP exposure [23]. Furthermore, throughout the various steps that RBCs undergo during donation and storage, several DAMPs may be generated. These include heme, eATP, HMGB1, and lipid peroxidation products [24]. In order to mitigate for storage lesions, it has been investigated whether transfusion of fresher RBC units can improve clinical outcomes. However, the ABLE study did not demonstrate improved clinical outcomes in critically ill patients receiving RBCs stored for seven days compared with receiving units stored for three weeks [25]. Similarly, the INFORM study aimed to determine whether the duration of red-cell storage affects mortality following transfusion in a general population of hospitalized patients. The study found no difference in mortality between patients receiving the freshest available units and those receiving the oldest available units [26]. Finally, the RECESS study (performed in patients undergoing cardiac surgery) also failed to demonstrate a clinical benefit of units stored up to 10 days compared with units stored for 21 days [27]. It must be emphasized that the clinical effects of perioperative transfusion are difficult to disentangle due to important confounding factors. These include the extent of blood loss, severity of pre-existing anemia, surgical complexity, the stage of the tumor and the overall patient frailty [28,29]. Each of these factors can independently affect postoperative outcomes.

These findings highlight the importance of patient blood management with an emphasis on the prevention and treatment of anemia while aiming to minimize blood loss in order to avoid unnecessary transfusion. When transfusion is clinically indicated, a restrictive transfusion strategy is generally favored in hemodynamically stable patients. The decision to transfuse should be guided by the patient’s overall clinical condition rather than hemoglobin concentration alone [30]. Thus, reducing exposure to allogeneic blood products may be preferable as a strategy for limiting transfusion-related immunologic and DAMP-mediated effects than preferentially selecting fresher RBC units.

**Figure 1:**
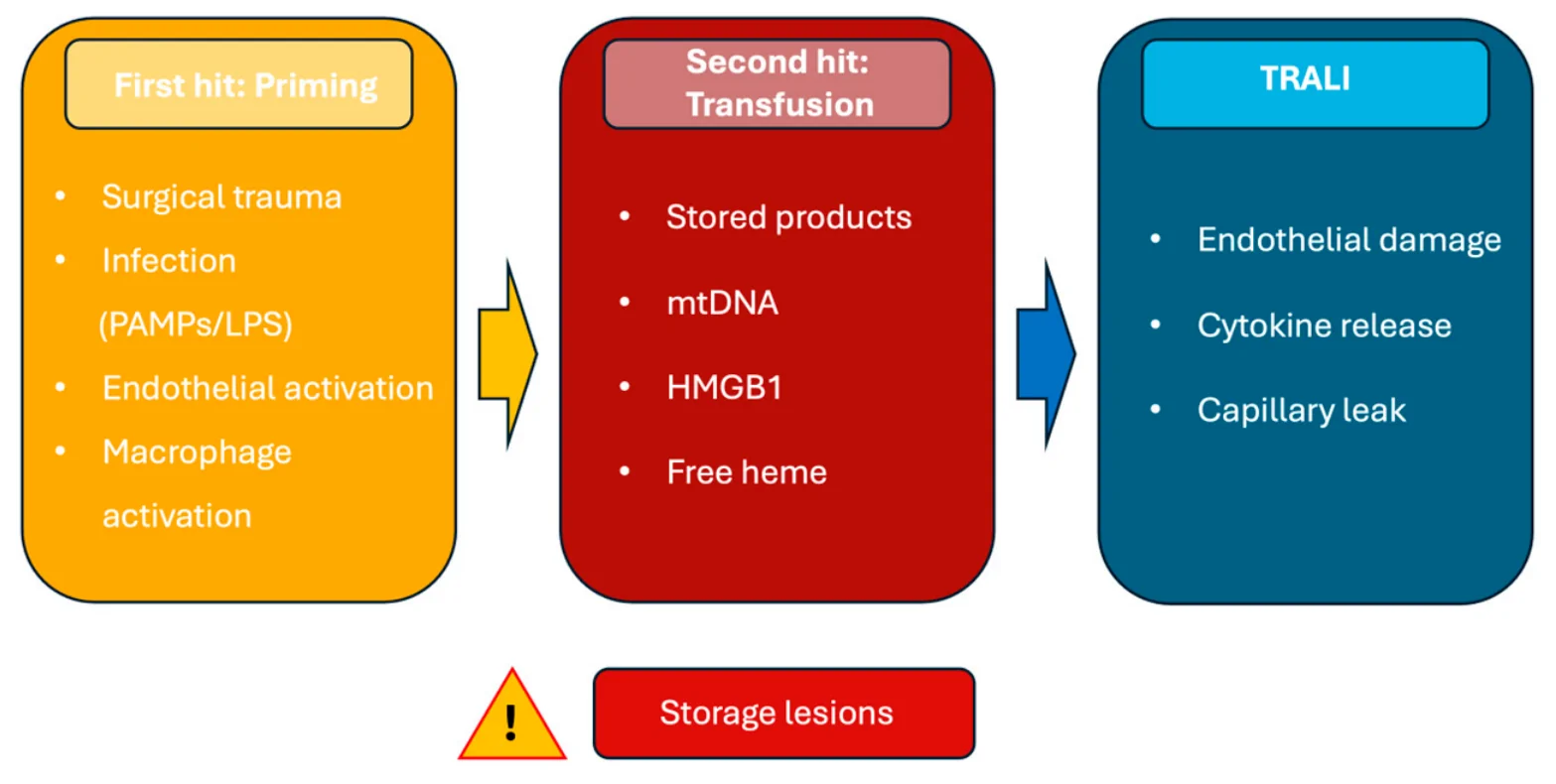
Two-hit model of TRALI in the perioperative setting. Abbreviations: TRALI, transfusion-related acute lung injury; PAMPs, pathogen-associated molecular patterns; LPS, lipopolysaccharide; mtDNA, mitochondrial DNA; HMGB1, high-mobility group box 1.

Importantly, transfusion-related acute lung injury (TRALI), a major adverse reaction associated with transfusion, is classified into TRALI Type I (without ARDS risk factors) and Type II (with an ARDS risk factor or pre-existing mild ARDS). Pathophysiologically, TRALI occurs via antibody-mediated pathways involving donor anti-HLA (Human Leukocyte Antigen) of Class I/II or anti-HNA (Human Neutrophil Antigen) antibodies, as well as non-antibody-mediated pathways. In this context, DAMPs generated during blood processing and storage act as possible contributors rather than the complete mechanism. These DAMPs seem to interact with PRRs of the host’s cells, including pulmonary endothelial cells and macrophages. It should be noted that the interaction between DAMPs and PRRs represents only the second hit in the two-hit hypothesis, which requires a preceding first hit. In experimental studies, LPS, the prototypical PAMP, has been used to initiate the first step, representing the priming phase, which can result in endothelial activation and neutrophil accumulation in the lung endothelium. The final steps of this process involve neutrophil activation, capillary leakage, and ultimately pulmonary edema [24]. The proposed two-hit mechanism underlying TRALI in the perioperative setting is illustrated in Figure 1. It should be noted that TRALI incidence has been markedly reduced due to the preferential use of male-predominant plasma and strategies targeting donor leukocyte antibodies [31,32]. However, evidence supporting leukoreduction as an independent TRALI-prevention strategy is not clear. Further studies before a definite conclusion can be reached are required [33].

Allogeneic transfusion is not the only approach when a patient’s clinical condition requires transfusion. Intraoperative autologous blood cell salvage (IOCS) represents an alternative that is classically debated during oncologic procedures. Available evidence, synthesized predominantly from observational studies, has not demonstrated worse oncologic outcomes associated with IOCS; however, the certainty of the evidence remains low [34]. In a small exploratory observational study of 17 patients undergoing major orthopaedic surgery, perioperative immune-cell profiles differed among patients receiving no transfusion, IOCS alone, or IOCS combined with allogeneic red blood cells. However, the study was not designed or powered to establish improved immune competence or clinical benefit from IOCS [35]. To the best of our knowledge, there are no studies investigating the presence of DAMPs in IOCS or potential differences in the type and quantity of DAMPs between IOCS and allogeneic transfusion. Thus, it remains unclear whether the different immunologic effects of IOCS and allogeneic transfusion are due to differences in DAMP exposure. Finally, it could be speculated that IOCS can be a source of PAMPs in cases where bacterial contamination takes place.

Far from being a neutral supportive measure, transfusion can exacerbate early immune cascades. When given during cancer surgery, transfusion-borne DAMPs may strike already primed tissue as a second hit, driving lung injury, immune dysfunction, and potentially poorer oncologic survival. Defining the clinical weight of this pathway will require targeted prospective studies. The main sources of perioperative DAMPs and mechanisms of release are summarized in Table 1.

## 4. Pharmacological Modulation of DAMPs in the Perioperative Setting

Unlike surgical trauma or transfusion, which directly introduce or release DAMPs and PAMPs through tissue injury or exogenous input, certain perioperative factors act through a fundamentally different mechanism. These are pharmacological modifiers that do not generate danger signals themselves but rather influence their release, sensing, and downstream signaling.

### 4.1. The Impact of Anesthesia on DAMPs

Anesthesia does not generate DAMPs in the same fashion as surgical incision, tissue disruption, or ischemia–reperfusion injury. Nevertheless, it is not a biologically neutral process. Instead of acting as a primary source of danger signals, anesthetic agents should be viewed as pharmacologic modifiers of DAMP release, sensing, and inflammatory signaling. This distinction is important in the perioperative setting, where the magnitude of sterile inflammation is not determined exclusively by surgical trauma. Anesthetic drugs can influence mitochondrial injury, oxidative stress, inflammasome activation, and PRR-mediated immune responses [36,37,38,39,40,41,42].

Among intravenous agents, propofol has been extensively studied in relation to HMGB1-mediated inflammatory signaling. Experimental studies have shown that propofol suppresses HMGB1 expression and release, reduces HMGB1-related mitochondrial oxidative injury, and attenuates NF-κB activity [37,38]. These effects are mechanistically relevant because HMGB1 and mtDNA are important perioperative DAMPs implicated in postoperative organ dysfunction [12,37,38,43].

Dexmedetomidine appears to exert similarly important modulatory effects on danger signaling. Preclinical studies have shown that dexmedetomidine attenuates inflammatory injury through inhibition of TLR4/NF-κB signaling, while more recent work suggests that dexmedetomidine may directly bind to and inhibit TLR4 [39,40].

Ketamine may also attenuate inflammatory signaling. However, DAMP-focused literature is less extensive. Existing evidence supports anti-inflammatory effects mediated mainly via suppression of NF-κB-related pathways, but its specific role in perioperative HMGB1 or mtDNA biology remains less clearly defined than that of propofol or dexmedetomidine [44].

The evidence regarding volatile anesthetics is more heterogeneous, with findings varying across studies. While some of the literature suggests anti-inflammatory or organ-protective effects in selected contexts, recent mechanistic data indicate that prolonged sevoflurane exposure may promote mtDNA-associated activation of the cGAS-STING-NLRP3 axis and postoperative neuroinflammation in experimental models. Therefore, volatile anesthetics should be best regarded as agents with context-dependent and potentially divergent effects on DAMP biology, rather than uniformly protective or deleterious drugs [41].

Clinical data on remimazolam remain limited. In a randomized study comparing remimazolam with propofol, levels of HMGB1 and NF-κB were not measured, while various postoperative markers of inflammation were higher in the remimazolam group. Therefore, it is not currently possible to establish a direct effect on DAMP regulation [42].

Thus, anesthetic drugs may influence perioperative danger signaling primarily by modifying DAMP release, sensing and downstream amplification rather than by independently driving inflammation. This perspective is more consistent with the concept of anesthesia as a perioperative immunologic modifier. It is particularly important in cancer surgery, where even subtle changes in innate immune activation may shape both short-term and long-term outcomes [36,45,46,47,48,49].

### 4.2. The Interplay Between Opioids and DAMPs

The relationship between opioids and perioperative danger signaling is still incompletely defined, with most available evidence supporting mechanistic plausibility and not direct clinical demonstration. Most of the available literature focuses on opioid-related immunomodulation and cancer outcomes in general, rather than on direct perioperative effects on DAMP release or DAMP sensing. Accordingly, current interpretations are based largely on mechanistic and preclinical studies, and caution is required when extrapolating these data to oncologic anesthesia practice [16,45].

Morphine is the best-described opioid in this context. Experimental studies indicate that repeated or chronic morphine exposure can promote HMGB1 release and HMGB1–TLR4/NF-κB-dependent microglial inflammation in models of opioid-induced hyperalgesia. These findings support a mechanistic link between morphine and DAMP-related TLR4 signaling, but they do not establish a perioperative or oncologic effect [50].

Evidence for fentanyl is more limited but clinically relevant. Recent experimental work suggests that fentanyl may not act as a strong primary TLR4 agonist on its own but can enhance LPS-induced TLR4/MD-2 signaling, NF-κB activation, and pro-inflammatory cytokine production in immune cells. This is important because, in the perioperative setting, fentanyl may therefore function less as an isolated trigger and more as an amplifier in the presence of concomitant PAMP exposure, such as endotoxemia, bacterial translocation, or occult infection [51].

By contrast, direct DAMP-focused perioperative data for remifentanil, sufentanil, and alfentanil is very limited. This knowledge gap deserves explicit acknowledgment. The absence of reliable data for these agents is especially important because they are widely used in modern anesthesia practice, yet they are often discussed by analogy with morphine or fentanyl despite a lack of agent-specific mechanistic or clinical evidence [45].

A major practical limitation is that perioperative opioid exposure is rarely attributable to a single drug. In real-world cancer surgery, patients often receive combinations of opioids throughout different perioperative phases, for example, fentanyl during induction, remifentanil intraoperatively, and morphine for postoperative analgesia. Therefore, attempts to assign a specific immunologic or oncologic effect to a single opioid are inherently confounded by mixed exposure, cumulative dose, timing of administration, co-administered anesthetics, surgical extent, transfusion, infection, and the use of regional techniques. These confounding factors likely contribute to the inconsistency of the broader literature linking perioperative opioids with cancer recurrence [45].

Overall, current evidence supports considering opioids as potential modifiers of perioperative danger signaling rather than generators of DAMPs. At present, morphine has the strongest mechanistic link to HMGB1- and TLR4-related signaling, and fentanyl has emerging evidence suggesting amplification of inflammatory responses under primed conditions. For several other commonly used perioperative opioids, the evidence base remains sparse. This uncertainty should be acknowledged explicitly in the review [16,52].

Collectively these findings show that anesthetic and/or analgesic drugs should be best seen as modifiers rather than primary determinants of perioperative danger signaling. Their immunologic impact seems to be related to surgical magnitude, ischemia–reperfusion, transfusion burden, microbial exposure, and baseline host immune status. Thus, the effect of anesthetic technique on DAMP/PAMP is likely to be context-dependent and modest relative to the inflammatory consequences of the operation itself.

## 5. Sensing Danger: Molecular Recognition and Signaling in the Perioperative Window

The biological effects of DAMPs and PAMPs are mediated through their recognition by PRRs, which are expressed on innate immune cells such as monocytes, macrophages, and neutrophils, as well as on endothelial and parenchymal cells. These receptors initiate intracellular signaling cascades that orchestrate inflammatory and immune responses in the perioperative setting.

Toll-like receptors (TLRs) are a class of PRRs capable of recognizing both endogenous and microbial ligands. TLR2 and TLR4 respond to multiple microbial products and have also been linked to the recognition of selected endogenous ligands. TLR9, in contrast, is an endosomal nucleic acid sensor that recognizes CpG-rich DNA after DNA-containing material has been internalized into endolysosomal compartments. Therefore, the measurement of bacterial DNA, cfDNA, or mtDNA in plasma should not be interpreted as an indication that TLR9 has been activated [9,53].

The receptor for advanced glycation end products (RAGE) acts in conjunction with TLRs to amplify selected inflammatory signals. The NLRP3 inflammasome is a cytosolic multiprotein complex that can respond to cellular stress signals, including P2X7 activation triggered by extracellular ATP, mitochondrial dysfunction, mtDNA, uric acid, and ROS. The cGAS-STING pathway differs from TLR9: cGAS detects DNA that has entered the cytosol, whereas TLR9 detects DNA within endosomal compartments. Circulating cfDNA or mtDNA may exist as free fragments, protein-bound complexes, or within extracellular vesicles, and its concentration in plasma does not indicate access to the cytosol, endosomal uptake, or activation of either pathway. Cellular transport, uptake, vesicular transport, and escape from endosomes are, therefore, critical mechanistic steps that should not be inferred solely from measurements of circulating DNA [53].

Taken together, these pathways form an interconnected but compartmentalized detection network. Perioperative measurements of circulating ligands provide evidence of exposure or release, while receptor activation and downstream signaling require separate mechanistic evidence.

## 6. Clinical Consequences of Perioperative Danger Signaling

The impact of perioperative DAMP and PAMP exposure depends not only on their release, but also on how these signals are sensed or amplified by the pathways of innate immunity. Understanding these molecular mechanisms links surgical injury to immune dysfunction and organ injury.

### 6.1. Early Postoperative Complications

DAMPs exert a dual role in the perioperative setting. At physiological concentrations, they may contribute to tissue repair, immune-cell recruitment, and wound healing. However, their release can promote sterile inflammation, endothelial dysfunction, coagulation abnormalities, and organ injury. This supports the concept of perioperative DAMP signaling as a plausible mechanistic link between surgical trauma and early postoperative complications [10,11,12,13,43].

Perioperative studies have linked circulating HMGB1, extracellular histones, and mtDNA with postoperative inflammatory responses and complications [12,13]. Mechanistic analyses further support a role for histones and HMGB1 in neutrophil extracellular trap (NET) formation [13]. DAMP-mediated mechanisms have also been implicated in postoperative pulmonary, renal, and neuroinflammatory complications; however, the strength and directness of evidence vary by organ system [6,43].

Human studies increasingly support the clinical relevance of circulating DAMPs after surgery. Plasma HMGB1 rises substantially after major surgery, and early postoperative increases in DAMPs and proteomic markers of innate immune dysregulation have been demonstrated in translational perioperative cohorts. Likewise, cell-free mtDNA has emerged as a candidate biomarker associated with inflammatory postoperative complications after cardiac surgery, suggesting that DAMP profiling may help identify patients at increased risk before overt clinical deterioration is apparent [11,12].

Overall, early postoperative complications should be viewed not only as consequences of surgical magnitude or comorbidity burden, but also as potential downstream manifestations of excessive perioperative danger signaling. This perspective supports further investigation of DAMPs as biomarkers of risk stratification and as potential mechanistic targets for perioperative organ protection [10,11,12,13,43].

### 6.2. Immunosuppression and Perioperative Immune Dysfunction

An important consequence of perioperative immune activation is dysregulation, rather than a simple linear transition from hyperinflammation to immunosuppression. Pro-inflammatory signaling and reduced immune function may overlap temporally, with reduced natural killer cell activity, reduced HLA-DR expression on monocytes, and reduced extracellular cytokine responses occurring in parallel with the continued release of inflammatory mediators. This concurrent phenotype may increase susceptibility to infections and delay recovery and is particularly significant following major colorectal and oncological surgeries [9,10,11,54].

Therefore, postoperative hyperinflammation and immunosuppression should not be treated as mutually exclusive conditions. The balance between inflammatory activation, tolerance, cellular exhaustion, and antimicrobial capacity varies among patients and over time, which limits the interpretation of any single DAMP or PAMP measurement as a surrogate for the overall immunological status.

Thus, perioperative danger signaling should be viewed not only as a mediator of sterile inflammation and organ injury, but also as a contributor to a transient window of perioperative immune vulnerability. Importantly, this perioperative immune landscape is not entirely fixed, as anesthetic and perioperative management strategies may influence the magnitude and direction of danger signaling responses. Selected effects of commonly used anesthetic approaches on DAMP-related pathways and immune modulation are summarized in Table 2. Randomized clinical evidence has also evaluated regional anesthesia in relation to cancer recurrence [55].

### 6.3. Oncological Implications of Perioperative Danger Signaling

In cancer surgery, transient perioperative immune vulnerability carries distinct oncologic significance. Surgical trauma, neuroendocrine activation, pain, blood transfusion, and DAMP signaling collectively suppress cytotoxic lymphocyte activity, impair antigen presentation, expand regulatory immune populations, and drive pro-angiogenic signaling [45,46].

This environment may facilitate survival of residual tumor cells or even micrometastases at a time when host immunosurveillance is weakened. Postoperative inflammation and immune dysfunction have been associated with angiogenesis, tumor-cell dissemination, and recurrence-promoting microenvironments, especially in colorectal and breast cancer [46,54].

From an anesthesiological perspective, perioperative management can influence these biological processes. Propofol-based anesthesia, regional techniques, and dexmedetomidine have shown potentially beneficial immunomodulatory effects in experimental and limited clinical settings; however, no anesthetic technique has been proven to decrease cancer recurrence, and the available oncological data remain contradictory [45,47,48,49,55].

At present, any link between perioperative danger signaling and oncologic outcomes should be viewed as supported by preclinical and mechanistic evidence rather than clinically established, with the strongest evidence supporting perioperative immune dysfunction in general, but less direct evidence linking specific perioperative DAMP signatures to recurrence or survival.

Therefore, perioperative danger signals should be considered not only as mediators of immediate postoperative morbidity, but also as biologically plausible contributors to long-term oncologic outcomes. This concept strengthens the rationale for integrating DAMP/PAMP biology into perioperative oncology research and exploring whether modulation of danger signaling may improve both short-term recovery and long-term cancer outcomes [46,54].

## 7. Perioperative Danger Signals: From Profiling to Clinical Translation

While the biology of perioperative danger signaling is increasingly clear, translating DAMPs and PAMPs into clinical practice remains challenging. Moving from bench to bedside requires standardized biomarker assays, robust detection protocols, and a practical strategy to overcome implementation barriers. Here, we synthesize current evidence and highlight emerging strategies designed to bridge this gap.

### 7.1. Biomarker Profiling of Perioperative Danger Signals

The concept of using DAMPs and PAMPs as biomarkers is not novel. For instance, serum HMGB1 has been proposed as a potential biomarker of kidney disease progression (n = 256; serum HMGB1 measured by ELISA; AUC = 0.892 for identifying diabetic kidney disease) [56] and of sepsis-induced organ failure (n = 60 patients with septic shock; HMGB1 measured before, immediately after, and 24 h after polymyxin B direct hemoperfusion; HMGB1 levels correlated with SOFA-defined organ failure) [57]. However, in the perioperative setting, the role of danger signals as biomarkers is largely underexplored. Given their association with postoperative complications, perioperative immune dysregulation, and potentially cancer recurrence, these signals could represent a valuable source of perioperative biomarkers.

At present, only a limited number of studies have examined circulating DAMPs in relation to postoperative complications. In a prospective study of 101 pediatric patients undergoing congenital cardiac surgery with CPB, plasma levels of HMGB1 and histones were significantly higher in neonates and infants who developed postoperative complications. DAMPs were assessed using proteomic analysis and ELISA, with blood samples collected at baseline, during CPB rewarming, upon ICU admission, and on first postoperative day. These findings demonstrate an association between elevated DAMP levels and postoperative complications [13]. However, they do not establish validated predictive performance.

Nevertheless, further study of DAMPs, possibly in the form of molecular signatures, is essential for cancer surgery with respect to cancer recurrence and early complications (ARDS, AKI and TRALI). Rather than serving as isolated markers of tissue injury, these molecules could be useful when interpreted as part of a perioperative signature. This signature could reflect the balance between surgical trauma, transfusion, immune activation and microbial exposure. In this context, danger signal profiling could offer a means of identifying patients at increased risk for adverse postoperative outcomes, particularly when biomarkers are used as integrated signatures rather than in isolation.

Representative human studies that directly evaluate perioperative DAMP biomarkers are summarized in Table 3. Overall, these studies support the notion that perioperative DAMP release is a reproducible biological phenomenon, while also highlighting significant diversity in patient populations, analytical methods, sampling timelines, and clinical endpoints.

### 7.2. Barriers to Clinical Translation

Despite the biological relevance regarding DAMP and PAMP profiling, investigation of these molecules remains largely confined to research settings. Most studied biomarkers, including HMGB1, histones, S100 proteins, and mtDNA, require laboratory-based assays such as immunoassays and nucleic acid quantification techniques, limiting their real-time perioperative applicability [58,59,60].

Substantial methodological difficulties remain, including variable sampling timepoints, unstandardized pre-analytical handling and assay, and a lack of validated clinical thresholds. These limitations are particularly important in perioperative medicine, where biomarker utility depends not only on analytical validity, but also on rapid turnaround and clinically actionable interpretation [61]. Analytical limitations are also biomarker-specific. HMGB1 measurements can be influenced by redox state, molecular complexing, serum-plasma differences and immunoassay interference [58,62], whereas cfDNA/mtDNA quantification is sensitive to haemolysis, platelet or leucocyte contamination, pre-analytical handling, primer selection and interference from nuclear mitochondrial sequences (NUMTs) [60].

Because these practical hurdles are currently unresolved, it should be stated that the clinical utility of perioperative DAMP and PAMP profiling is largely hypothetical.

However, mechanistic plausibility does not always translate into clinical benefit, as targeted interventions directed at danger-sensing pathways did not show improved outcomes in critical-illness settings. The ACCESS randomized trial with the objective to determine a reduction in sepsis-induced mortality using a TLR4 antagonist (eritoran) did not demonstrate a reduction in 28-day mortality [63]. Similarly, another TLR4 inhibitor (TAK-242) was evaluated as a potential pharmacological suppressor of cytokine levels and its effect on 28-day all-cause mortality in patients with sepsis. TAK-242 failed to suppress cytokine levels in this patient population and mild increases in serum methemoglobin were also observed [64].

From a practical perspective, the development of rapid point-of-care assays represents an essential step toward the clinical application of DAMP/PAMP profiling. The COVID-19 pandemic highlighted the importance of rapid point-of-care diagnostics [65], comparable tools are not currently available for perioperative DAMP/PAMP assessment. Therefore, although danger signal profiling holds translational promise, routine clinical implementation remains unlikely unless these barriers are addressed.

### 7.3. Role of Artificial Intelligence and Future Directions

Perioperative danger signaling encompasses DAMPs, PAMPs, and PRRs, which are all modulated by interacting perioperative variables. These variables can include the chosen anesthetic plan, the magnitude of the surgery itself, and blood transfusion. Relying on a sole biomarker does not suffice to provide accurate risk stratification regarding postoperative complications and perioperative immune dysfunction.

**Figure 2:**
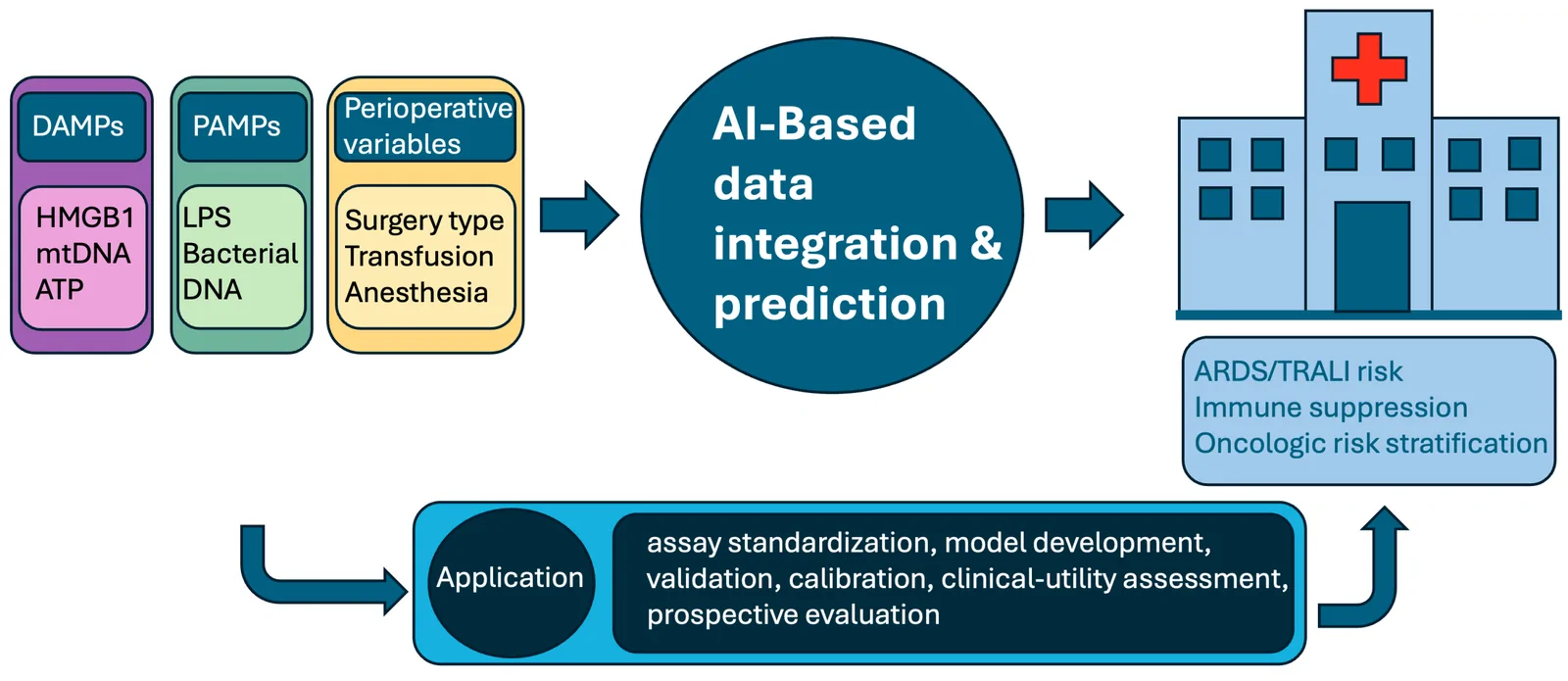
Proposed framework for the development and clinical evaluation of AI-assisted perioperative DAMP/PAMP profiling. Colored boxes represent DAMPs (purple), PAMPs (green), and perioperative variables (yellow), while arrows indicate the proposed workflow from data integration and prediction to clinical application. Abbreviations: DAMPs, damage-associated molecular patterns; HMGB1, high-mobility group box 1; mtDNA, mitochondrial DNA; ATP, adenosine triphosphate; PAMPs, pathogen-associated molecular patterns; LPS, lipopolysaccharide; ARDS, acute respiratory distress syndrome; TRALI, transfusion-related acute lung injury.

AI has the potential to analyze high-dimensional data and identify composite biomarker signatures rather than relying on isolated molecular markers [66]. This is important in DAMP/PAMP biology, where individual biomarkers may lack specificity. In a future research setting, integrated molecular signatures combined with clinical variables could potentially improve risk prediction and help identify informative biomarker combinations, supporting biomarker development [67,68]. As illustrated in Figure 2, such an approach could provide a framework for integrating perioperative molecular and clinical data.

However, the clinical utility of AI-assisted DAMP/PAMP profiling remains hypothetical. Major barriers include the lack of large and uniform datasets, biomarker harmonization, and explainability of “black-box” predictions in high-stakes perioperative decision-making [69,70,71]. Accordingly, AI-assisted DAMP and/or PAMP profiling should currently be considered as a future research concept and not as an emerging clinical tool. Important methodological and clinical validation would be required before such approaches could be considered for routine perioperative application.

### 7.4. Limitations

Several limitations of the present review and the underlying evidence should be acknowledged. Firstly, this article is structured as a narrative review rather than a systematic review. Thus, formal risk-of-bias assessments, protocol pre-registration, and meta-analytic evidence grading were not performed. Secondly, the available literature shows a marked predominance of preclinical and in vitro experimental models. Translating these pathways directly to complex clinical settings is confounded by anesthesia involving multiple agents, surgical variability, and patient baseline states. Thirdly, substantial assay heterogeneity and variable sampling timepoints, along with the absence of standardized pre-analytical handling protocols, limit the reproducibility of DAMP and PAMP profiling. Finally, direct clinical evidence that links specific perioperative danger signatures to oncologic outcomes (including recurrence) remains scarce and associative.

## 8. Conclusions

Danger signals during the perioperative window mediate the relationship between surgical stress, inflammation, and immune dysregulation. DAMP profiling and potentially selected PAMP-related markers may contribute to perioperative risk stratification and may clarify mechanisms driving postoperative complications. A link between perioperative danger signals and long-term cancer outcomes makes biological sense, but clinical proof is still lacking. Key priorities include establishing robust biomarker panels, tuning predictive algorithms, and profiling the specific DAMP load in intraoperative cell salvage to explain its distinct immune signature compared with allogeneic blood. Integrating danger-signal biology into perioperative research may help evaluate its potential role in risk stratification, organ protection, and perioperative oncologic care, while supporting further development of the emerging field of onco-anesthesia.

## Figures and Tables

**Table 1:** Sources of Perioperative DAMPs, PAMPs and Mechanisms of Release.

Perioperative Factor	Mechanism of Injury	Major Danger Signal Released	Clinical Context	Ref.
Surgical trauma	Tissue disruption, necrosis, cauterization	HMGB1, histones, ATP, mtDNA, HSPs, S100A8/A9,	All major surgeries	[9,10,11,13]
Ischemia–reperfusion	ROS production, ATP depletion, regulated cell death (pyroptosis, necroptosis)	HMGB1, mtDNA, ATP	Vascular, transplant, cardiac surgery	[7,9,12,13]
Cardiopulmonary bypass	Blood contact with artificial surfaces, ischemia	cf-mtDNA, HMGB1, histones, S100A8/A9	Cardiac surgery	[12,13]
Blood transfusion	Storage lesion, hemolysis, microparticles	Free heme, cfDNA, ATP, HMGB1	Major surgery, trauma	[23,24]
Mechanical ventilation	Alveolar stretch (volutrauma)	ATP	Experimental ventilator-induced lung injury models	[14]
Infection (PAMPs)	Microbial invasion	LPS, peptidoglycan, bacterial DNA	Sepsis, surgical infection	[15,16]

Abbreviations: PAMPs, pathogen-associated molecular patterns; HMGB1, high-mobility group box 1; ATP, adenosine triphosphate; mtDNA, mitochondrial DNA; HSPs, heat shock proteins; S100A8/A9, S100 calcium-binding protein A8/A9; ROS, reactive oxygen species; cfDNA, cell-free DNA; LPS, lipopolysaccharide.

**Table 2:** Potential modulation of perioperative danger signaling by common anesthetic.

Agent/Technique	Evidence Level	Direct Agent-Specific Evidence	Clinical Interpretation and Key Limitations	Ref.
Propofol	In vitro + animal	Reduced HMGB1 expression/release and NF-κB activity in LPS-stimulated macrophages; reduced stretch-induced HMGB1 release and HMGB1-related mitochondrial oxidative injury in experimental lung models.	Supports a preclinical HMGB1-modulating effect. No randomized human perioperative DAMP trial in these studies; mtDNA reduction and oncologic benefit were not directly demonstrated.	[37,38]
Dexmedetomidine	Animal + in vitro/translational	Reduced TLR4/NF-κB signaling in experimental injury models; direct binding to and inhibition of TLR4 demonstrated with reporter/binding assays, with reduced microglial activation in vitro and in vivo.	Mechanistic evidence for TLR4 modulation, but predominantly preclinical. Direct perioperative human DAMP outcomes and oncologic benefit remain unproven.	[39,40]
Sevoflurane	In vitro and Animal model	Prolonged exposure promoted mitochondrial fission, cytosolic mtDNA release, cGAS-STING activation, and NLRP3 inflammasome activation in mice and microglia.	Suggests context-dependent pro-inflammatory DAMP signaling in experimental neuroinflammation. The mouse/cell model and prolonged exposure cannot be generalized to routine clinical anesthesia or cancer recurrence.	[41]
Ketamine	In vitro and animal	Reduced LPS-induced HMGB1 release and inhibited NF-κB/p38 MAPK signaling in macrophages; reduced HMGB1 and organ injury in a rat sepsis model.	Preclinical evidence of HMGB1-pathway modulation. These are sepsis models, not perioperative cancer surgery, and there is no human perioperative DAMP validation.	[44]
Remimazolam	Randomized human trial	HMGB1 and NF-κB were not measured. Versus propofol, remimazolam was associated with higher CRP, IL-6, leukocyte and neutrophil counts at 24 h; TNF-α and S100β did not differ.	Does not support a direct DAMP-modulating effect. Single-center RCT (*n* = 92) in older VATS patients; comparator was propofol and no DAMP-specific assay was performed.	[42]
Morphine	In vitro and animal model	Chronic morphine exposure increased HMGB1-TLR4/NF-κB-related microglial inflammatory signaling in models of opioid-induced hyperalgesia.	Agent-specific mechanistic link to HMGB1-TLR4 signaling, but the chronic hyperalgesia model does not represent perioperative dosing and does not establish postoperative or oncologic effects.	[50]
Fentanyl	In vitro/ex vivo	Enhanced LPS-induced TLR4/MD-2 signaling and pro-inflammatory mediator responses in rat microglia and human monocyte-derived macrophages; NF-κB activation was demonstrated under LPS-primed conditions.	May amplify inflammatory signaling when a PAMP stimulus is present. No direct perioperative human DAMP study or demonstrated cancer-recurrence effect.	[51]
Regional anesthesia-analgesia	Randomized human trial	Paravertebral block + propofol versus sevoflurane + opioid analgesia: breast-cancer recurrence was similar (HR 0.97, 95% CI 0.74–1.28; *p* = 0.84). DAMPs were not measured.	No established oncologic protection. The trial compared bundled anesthetic strategies, so the independent effect of regional anesthesia cannot be isolated.	[55]

Abbreviations: DAMPs, damage-associated molecular patterns; HMGB1, high-mobility group box 1; mtDNA, mitochondrial DNA; NF-κB, nuclear factor kappa B; LPS, lipopolysaccharide; TLR4, Toll-like receptor 4; MD-2, myeloid differentiation factor 2; PAMP, pathogen-associated molecular pattern; HR, hazard ratio; CI, confidence interval; cGAS-STING, cyclic GMP-AMP synthase–stimulator of interferon genes; NLRP3, NOD-, LRR- and pyrin domain-containing protein 3; MAPK, mitogen-activated protein kinase; IL-6, interleukin-6; CRP, C-reactive protein; TNF-α, tumor necrosis factor alpha; S100β, S100 calcium-binding protein beta; RCT, randomized controlled trial; VATS, video-assisted thoracoscopic surgery.

**Table 3:** Key human studies evaluating perioperative DAMP biomarkers and clinical outcomes.

Population	Sample Size	Biomarker(s)	Assay	Sampling Time	Main Outcome	Key Limitations	Ref.
Adults undergoing cytoreductive surgery + HIPEC	n = 20	HMGB1, HSP70, S100A8/A9, S100A12, nDNA, mtDNA	ELISA; qPCR	Baseline; After CRS; after HIPEC; ICU admission; POD1	Significant DAMP release and immunosuppression were observed; the peak HMGB1 level was higher in patients who later developed a postoperative infection.	Small, single-center observational cohort; only five cases of infection; no validated predictive threshold.	[10]
Adults undergoing elective colorectal surgery	n = 100	HMGB1, HSP70, nDNA, mtDNA	ELISA; qPCR; exploratory Olink proteomics and ATAC-seq	Preoperative; end of surgery; POD1; POD3 when available	Levels of HMGB1, nDNA, and mtDNA increased following surgery; the postoperative increase in DAMP was accompanied by reduced cytokine production ex vivo and molecular changes in monocytes.	A substudy of a randomized controlled trial (RCT) that was not primarily designed to identify biomarkers; exploratory analyses of genomic data in smaller subgroups; clinical prognostic performance has not been established.	[11]
Older adults undergoing total hip arthroplasty	n = 287	HMGB1, IL-6, IL-1β, TNF-α, CRP	ELISA	Preoperative; 24, 48 and 72 h postoperative	HMGB1 levels were higher in patients with postoperative delirium and were associated with inflammatory cytokines; HMGB1 was identified as a potential prognostic biomarker.	A single-center observational study; correlation does not prove causation; findings specific to a particular process and a particular population.	[43]
Adults undergoing surgical aortic valve replacement with CPB	n = 24	Cell-free mtDNA and fragment size; inflammatory markers	qPCR-based cf-mtDNA quantification; leukocyte/plasma inflammatory profiling	Baseline; intraoperative; 6, 12, 18 h after ICU admission	cf-mtDNA increased approximately 16-fold at the end of CPB; exploratory analyses showed only non-significant trends toward associations with postoperative bleeding, infection, hepatic failure, and hospital length of stay.	Pilot, single-center study; none of the exploratory outcome associations reached conventional statistical significance; external validation is required.	[12]
Pediatric patients undergoing congenital cardiac surgery with CPB	n = 101	Histones, HMGB1, S100A8/A9, and other DAMPs; NET related markers	Proteomics; ELISA; neutrophil transcriptomic analyses	Serial perioperative sampling including baseline, end of CPB, ICU admission, and POD1	DAMPs and neutrophil extracellullar traps were elevated in patients with postoperative complications; histones and HMGB1 showed increased levels in the group with complications.	A heterogeneous age range among the children and surgical complexity; mechanistic analyses based in part on subgroups; absence of a validated clinical cutoff or external validation.	[13]

Abbreviations: CPB, cardiopulmonary bypass; DAMPs, damage-associated molecular patterns; HMGB1, high-mobility group box 1; mtDNA, mitochondrial DNA; cf-mtDNA, cell-free mitochondrial DNA; HSP70, heat shock protein 70; S100A8/A9, S100 calcium-binding protein A8/A9; S100A12, S100 calcium-binding protein A12; nDNA, nuclear DNA; NET, neutrophil extracellular trap; IL-6, interleukin-6; IL-1β, interleukin-1 beta; TNF-α, tumor necrosis factor alpha; CRP, C-reactive protein; RCT, randomized controlled trial; HIPEC, hyperthermic intraperitoneal chemotherapy; CRS, cytoreductive surgery; ELISA, enzyme-linked immunosorbent assay; qPCR, quantitative polymerase chain reaction; ICU, intensive care unit; POD1, postoperative day 1; ATAC-seq, assay for transposase-accessible chromatin using sequencing.

## Data Availability

No new data were created or analyzed in this study. Data sharing is not applicable to this article.
